# EXPLAINING REHABILITATION AS AN INTERVENTION TO STUDENTS, PATIENTS, AND THE PUBLIC: THE “REHABILITATION CHOCOLATE CAKE”

**DOI:** 10.2340/jrm.v58.46458

**Published:** 2026-08-24

**Authors:** Stefano NEGRINI, Carlotte KIEKENS

**Affiliations:** 1 Department of Biomedical, Surgical and Dental Sciences, University of Milan, Milan; 2 Laboratory of Evidence-Based Medicine, IRCCS Galeazzi-Sant’Ambrogio Hospital, Milan, Italy

## REHABILITATION AS AN INTERVENTION

Rehabilitation is a valuable concept. Unfortunately, it is used for many purposes, both within and outside the health sector, which poses challenges for clinicians who primarily deal with rehabilitation. Previous studies showed the confusion surrounding the term in medicine overall (1), among the public on Google (2), and for researchers in PubMed and Cochrane Reviews (3). The recently published reporting Guideline for Intervention Description in Rehabilitation – GUIDE-Rehab (4) – has identified at least 4 different contexts where the single term rehabilitation is used within medicine, each with different meanings and definitions: to describe a health strategy (5), a health sector (6), a health workforce (7), and a specific health intervention (8). In this research letter, we focus only on the intervention as defined by the Cochrane Rehabilitation, Functioning, and Disability thematic group (CochraneRehab) (8).

## REHABILITATION DEFINITION

To address this issue, CochraneRehab established a specific initiative several years ago that resulted in a definition based on the Population, Intervention, Outcome (PIO) research framework and the International Classification of Functioning, Disability and Health (8). The definition states: “In a healthcare context, rehabilitation is a multimodal, person-centred, collaborative process, including interventions targeting a person’s capacity (by addressing body structures, functions, and activities/participation) and/or contextual factors related to performance, to optimise the functioning of persons with health conditions currently experiencing disability or likely to experience disability, or persons with disability” (8). It is founded on widely accepted concepts related to the Outcome (optimizing functioning) and the Population (people with health conditions who experience disability). The Intervention section is divided into 2 parts: specific, focusing on how the intervention is delivered (with emphasis on capacity and contextual factors related to performance), and general, which is the newest and requires more explanation for all audiences.

The Intervention general section focuses on 4 terms, which could be explained as follows:

- Multimodal: application of more than 1 intervention or an intervention with multiple components.
- Person-centred: interventions are chosen and tailored to an individual’s needs and engagement, building on and strengthening the person’s resources while considering their values, preferences, goals, and contextual factors.
- Collaborative: participation of the individual(s) providing the interventions and those engaged in rehabilitation. The level of participation and the participants vary according to health condition(s), rehabilitation phase (acute, post-acute, chronic), and contextual factors, including setting(s) (inpatient, outpatient, home, community).
- The process involves 1 or more consecutive rehabilitation cycles, including assessment, goal setting, assignment, interventions, evaluation, and repetition, if necessary, until optimal functioning is achiev-ed. This is commonly known as the Rehab-Cycle.

Not all these terms are intuitive and easy to under-stand. Analogies are a useful way to convey complex information. To enhance students’ and the wider rehabilitation audience’s understanding of these concepts across various contexts, we developed an analogy to explain these terms, which was well received and has proved highly effective at several scientific meetings and congresses. We summarize it here for others to use.

## THE “REHABILITATION CHOCOLATE CAKE”

**Fig. 1:**
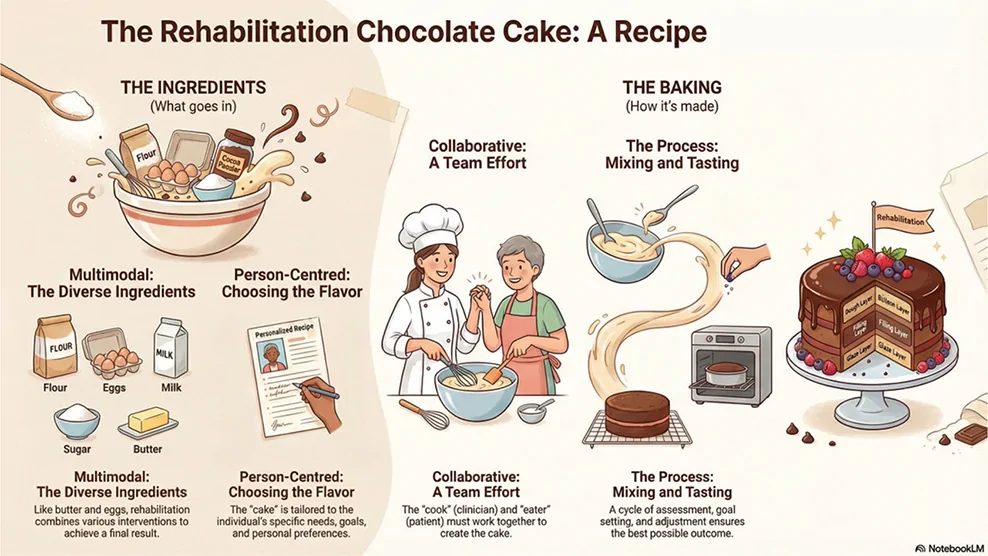
The “rehabilitation chocolate cake”.

To make a good chocolate cake, as shown in Fig. 1, you should focus on:

- Initial ingredients (butter, chocolate, eggs, yeast, flour, salt, sugar): the different components you combine to achieve the final result. These are like all the interventions we assemble when providing rehabilitation. It is the multimodal concept.
- Which cake does the client want? In practice, the choice is partly yours and partly that of the final eaters. You know the ingredients and how to mix them but, in the end, what matters is whether the final product will be appreciated. It is the person-centredness concept.
- How to determine the ingredients in a team. The rehabilitation cake is created through a team effort, including the eater(s), who must actively participate alongside the cook(s). This is the collaborative concept.
- Finally, you can make the cake by accurately measuring and mixing the ingredients, with correct quantities, proportions, sequence of steps, and timing. You can also assess (taste) and adjust as necessary or preferred. This human behavioural factor plays a crucial role in the final outcome: there are skilled and unskilled cooks, and similarly, good and bad cakes. It illustrates the process concept, including the rehab cycle.

## THE “REHABILITATION CHOCOLATE CAKE” TO UNDERSTAND GUIDE-REHAB

This analogy also works well with the recently published Guideline for Intervention Description in Rehabilitation (GUIDE-Rehab) (4). In fact, to describe the rehabilitation process, GUIDE-Rehab includes the ingredients (again, eggs, chocolate, etc.), which are different and mixed in various ways to produce the components (like the dough, the glaze, and the filling) that, together, form the final product (the cake) (see Fig. 1). The component theory and the intervention theory describe the processes (the recipes) for creating the components and the final cake, respectively.

## CONCLUSION

The intervention “rehabilitation” has been defined (8), and its characteristics can be described in detail (4). Implementing these methodological advances requires a deep understanding of their meanings, particularly when explaining them to the public or educating students. Examples can foster understanding and support subsequent implementation. The “rehabilitation chocolate cake” serves as a tool to define the scope and could advance scientific progress in the field.
